# Peptide conformational imprints enhanced the catalytic activity of papain for esterification

**DOI:** 10.3389/fbioe.2022.943751

**Published:** 2022-08-16

**Authors:** Kiran Reddy Kanubaddi, Ching-Lun Yang, Pei-Yu Huang, Chung-Yin Lin, Dar-Fu Tai, Chia-Hung Lee

**Affiliations:** ^1^ Department of Life Science and Institute of Biotechnology, National Dong Hwa University, Hualien, Taiwan; ^2^ Department of Chemistry, National Dong Hwa University, Hualien, Taiwan; ^3^ Medical Imaging Research Center, Institute for Radiological Research, Chang Gung University, Taoyuan, Taiwan; ^4^ Department of Neurology, Chang Gung Memorial Hospital, College of Medicine, Chang Gung University, Taoyuan, Taiwan

**Keywords:** esterification, molecularly imprinted polymers, papain, peptide conformational imprints, enzyme immobilization

## Abstract

Peptide conformational imprints (PCIs) offer a promising perspective to directly generate binding sites for preserving enzymes with high catalytic activity and stability. In this study, we synthesized a new chiral cross-linker cost-effectively for controlling the matrix morphology of PCIs on magnetic particles (PCIMPs) to stabilize their recognition capability. Meanwhile, based on the flank part of the sequences on papain (PAP), three epitope peptides were selected and synthesized. Molecularly imprinted polymers (MIPs) were then fabricated in the presence of the epitope peptide using our new cross-linker on magnetic particles (MPs) to generate PCIMPs. PCIMPs were formed with helical cavities that complement the PAP structure to adsorb specifically at the targeted position of PAP. PCIMPs^65–79^ were found to have the best binding parameters to the PAP with *K*_d_ = 0.087 μM and *B*_max_ = 4.56 μM. Upon esterification of *N*-Boc-His-OH, proton nuclear magnetic resonance (^1^H-NMR) was used to monitor the yield of the reaction and evaluate the activity of PAP/PCIMPs. The kinetic parameters of PAP/PCIMPs^65–79^ were calculated as *V*_max_ = 3.0 μM s^−1^, *K*_m_ = 5 × 10^−2^ M, *k*_cat_ = 1.1 × 10^–1^ s^−1^, and *k*_cat_/*K*_m_ = 2.2 M^−1^ s^−1^. In addition, PAP is bound tightly to PCIMPs to sustain its activity after four consecutive cycles.

## Introduction

Papain (PAP) is a cysteine protease (EC 3.4.22.2) found in papaya tease. Its substrate contains arginine or lysine residue and is commonly used in the food industry. PAP acts as a highly specific and effective biocatalyst and has been reported to catalyze carbon–carbon formation in organic synthesis (Cajnko et al., 2020; Cajnko et al., 2021; Bjelić et al., 2022). It operated under mild reaction conditions and separated easily from the reaction mixture (Morcelle et al., 2006; Jeong et al., 2011; Llerena-Suster et al., 2012; Cao et al., 2015). PAP-mediated esterification has been studied previously. It decreases the environmental impact of chemical alterations that typically require acyl chlorides or toxic coupling agents. In fact, PAP is usually utilized for trans-esterification of alkyl or vinyl esters in a medium with low water content (Prabhakar et al., 2017; Marathe et al., 2020). Moreover, PAP was also found to possess esterase activity in a biphasic system or an aqueous solution, and *N*-Boc amino acid esters were synthesized (Cantacuzène et al., 1987; de Beer et al., 2012). PAP shows remarkable catalytic performance in esterification, transesterification, and hydrolysis (Jeong et al., 2011; Anwar et al., 2017), but some of its properties may not fit industrial requirements due to inherent limitations such as lack of reusability, instability in organic solvents at high temperatures, and denaturing at different pH ranges (Homaei et al., 2010; Sheldon, 2011; Homaei, 2015).

Over the last five decades, numerous methods have been developed to immobilize all classes of enzymes. Enzyme immobilization technology was developed to reduce drawbacks and make them reusable at a commercial level (Homaei et al., 2013; Mohamad et al., 2015; Liang et al., 2021). Immobilized enzymes as catalysts were reviewed (Basso and Serban, 2019; Kankala et al., 2019). Previously, Tai and colleagues immobilized PAP on Sephadex G-50 to convert *N*-protected amino acids to their methyl esters (Tai et al., 1989). Since then, many efforts have been dedicated to utilizing PAP as a biocatalyst for diverse catalytic applications. For example, Storer and colleagues immobilized PAP on celite (PAP/celite) to catalyze the esterification of Cbz-glycine with methanol as a substrate in 12 different solvents of widely varying polarity (Stevenson and Storer, 1991). *N*-Cbz-L-alanine was converted to its corresponding esters with 2-phenethyl alcohol by using PAP/celite (Shih et al., 1997). Although these techniques can improve enzyme stability, they might also be accountable for restrictions in local and global protein flexibility. Inevitably, protein conformation was unstable under harsh synthesis conditions (Secundo, 2013; Hoarau et al., 2017). Enzymes lose their structure orientation during immobilization processes, which plays a crucial role in reducing enzymatic activity (Basso and Serban, 2019; Nguyen et al., 2019).

**FIGURE 1:**
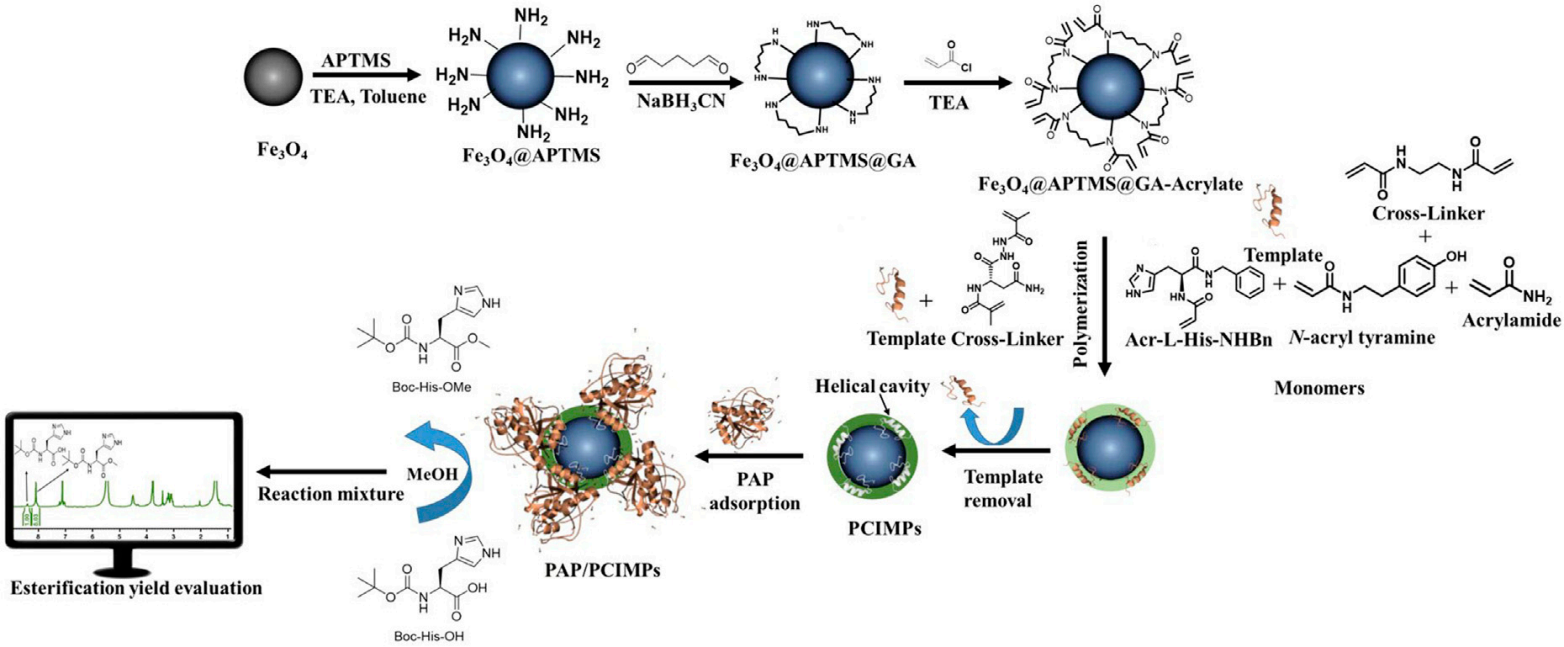
Schematic diagram of the (Top) fabrication of MPs to PCIMPs using cross-linker, monomers, and template (Bottom) application of PCIMPs using their binding mechanisms toward PAP and PAP/PCIMPs catalyzed esterification and yield evaluation under the monitor. APTMS, 3-aminopropyl trimethoxysilane; TEA, trimethylamine; GA, glutaraldehyde; MPs, magnetic particles; PAP, papain; PCIs, peptide conformational imprints; PCIMPs, peptide conformational imprint magnetic particles.

The fabrication of peptide conformational imprints (PCIs) on magnetic particles (PCIMPs) is a delicate process to immobilize enzymes (Chou et al., 2020; Kanubaddi et al., 2021). A helical peptide fragment of the enzyme was used as a template to form molecularly imprinted polymers (MIPs). As helical peptide segment-mediated PCIMPs were constructed, elegant helical cavities complementary to the enzyme structure can be achieved (Chou et al., 2020; Kanubaddi et al., 2021). By taking this dynamic property into account, the selection of the template (Figure 1) was crucial to generate helical cavities using PCIs. Binding enzymes tightly at the target position is the key issue in keeping enzymes flexible as well as stable. The advantage of this method is to create accessible binding sites on the surface of MPs and enable *catalytic active sites* with less interference during catalysis. The novelty of this work is to monitor the esterification yield of Boc-His-OH directly using ^1^H-NMR with great accuracy; demonstrate the activation effect of PCIMPs on the esterification activity of PAP; and sustain the esterification process of PAP/PCIMPs for four consecutive cycles. Finally, the immobilized enzyme is contemplated to further increase the efficiency and convenience in the catalysis of the reverse reaction of hydrolysis.

Our research methods can be divided into four main categories: 1) synthesis of a cross-linker and monomers from asparagine; 2) fabrication of PCIMPs using a single cross-linker; 3) adsorption of PAP to PCIMPs to obtain binding parameters; 4) operation of PAP/PCIMP-catalyzed esterification to observe enzyme kinetics.

## Materials and methods

The synthesis of the monomers is described in our previous studies. Briefly, Acr-L-His-NHBn (Tai et al., 2011) was prepared using the following steps. Initially, Boc-L-His-OMe was synthesized using Boc-His-OH and methanol by adding PAP as a catalyst (Tai et al., 1989). Then, the obtained Boc-L-His-OMe was converted to Boc-L-His-NHBn using benzylamine in the presence of PAP. Next, trifluoroacetic acid was used to deprotect the Boc group to form *L*-His-NHBn (Tai, 2003); then, acrylation of *L*-His-NHBn was carried out to obtain Acr-L-His-NHBn. Finally, another monomer, *N*‐acryl tyramine, was prepared by acylation with acrylic chloride of tyramine hydrochloride (Singh et al., 2013).

### Synthesis of a cross-linker (Metha-Asn-NHNH-Metha)

**SCHEME 1:**
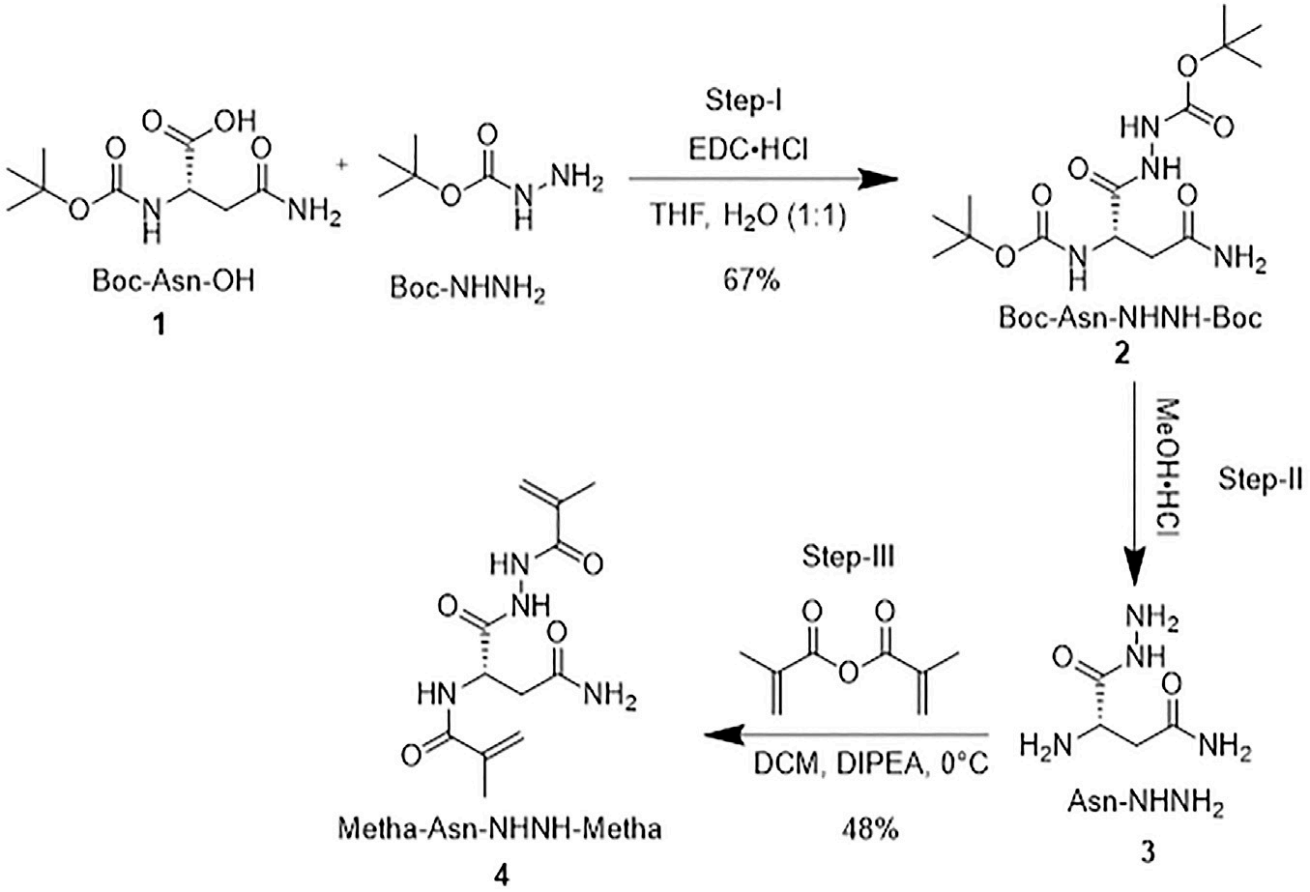
Synthesis of a cross-linker.

Scheme 1 shows the synthetic route of the cross-linker. Briefly, a mixture of Boc-L-Asn-OH (1 g, 1 eq) and Boc-NHNH_2_ (0.68 g, 1.2 eq) was dissolved in a water (H_2_O)/tetrahydrofuran (THF) mixture (1:1) and stirred for a few minutes. Later, 3-(ethyliminomethyleneamino)-*N,N*-dimethyl-propan-1-amine hydrochloride (EDC⋅HCl; 0.828 g, 1 equiv) was added portion-wise to the solution, stirred for 4 hours, and monitored by thin layer chromatography (TLC). The solution was extracted into ethyl acetate (EtOAc, 10 ml) and washed with 0.1 N hydrochloric acid (HCl), followed by H_2_O and saturated brine solution. The organic layer was passed through sodium sulfate (Na_2_SO_4_). The solvent was removed under a rotary evaporator to obtain **2** as a white solid (1.0 g, 67%). Boc-L-Asn-NHNH-Boc **2** (1 g) was dissolved in 10 ml of methanolic HCl (4 M). The mixture was stirred for 4 h at 0°C. After that, the solvent was evaporated under the rotary evaporator at room temperature (RT) and washed three times with diethyl ether (Et_2_O, 10 ml) to form a precipitate. The solid was dried under vacuum at 0°C to obtain 0.5 g of *L*-Asn-NHNH_2_ **3**. It was then dissolved in 10 ml of dry dichloromethane (DCM) and flushed with nitrogen (N_2_). Methacrylic anhydride (2 equiv.), followed by *N*,*N*-diisopropylethylamine (DIPEA; 5 equiv) were added dropwise and stirred overnight at 0°C under N_2_. Finally, the solution was extracted into DCM. The organic layer was washed with 0.1 N HCl, followed by H_2_O and saturated brine solution. The organic layer was mixed with anhydrous Na_2_SO_4_ to remove moisture and was purified by column chromatography. A solid was precipitated with a mixture of DCM and hexane solvents, which was further dried at 0°C and stored at −5°C. In this way, cross-linker **4** was obtained with a 48% yield (see the entire process in Supplementary Figure S2).

### Synthesis of the template

The peptide segments, such as PAP^65–79^ (GGYPW SALQLVAQYG), PAP^65-78^ (GGYPWSALQLVAQY), PAP^66-79^ (GYPWSALQLVAQYG), and PAP^66-78^ (GYPWSALQLVAQY) were synthesized using solid-phase chemistry of the fluorenylmethoxycarbonyl (Fmoc) method (Collins et al., 2014). The template (peptide residues) was synthesized using a CEM Discover Microwave-assisted Peptide synthesizer (Kohan Co., Taipei, Taiwan) as described previously (Collins et al., 2014; Lin et al., 2019; Kanubaddi et al., 2021). Subsequently, the purity of these peptides was monitored by HPLC equipped with an RP-18, using a mobile phase of 75:25 v/v methanol/water at RT and at a flow rate of 1 ml/min. Sharp peaks were observed at a retention time of ∼19 min, known as a template (Supplementary Figure S1A); these peptides’ purity was observed to be ∼88%. The template samples such as PAP^65–79^, PAP^65–78^, and PAP^66–79^ were analyzed using a Shimadzu Matrix-Assisted Laser Desorption/Ionization-Time of Flight (MALDI/TOF) mass spectrometer (MS) (Kyoto, Japan), and PAP^66-78^ (GYPWSALQLVAQY) was analyzed using Bruker Autoflex MALDI/TOF mass spectrometry (Germany) with 2,5-dihydroxybenzoic acid (DHB) as the matrix. of The sharp peaks of PAP^66-78^ were observed in the HPLC chromatogram at a retention time of ∼3 min, and purity was observed to be ∼95% (Supplementary Figure S1C). The reported *m/z* values of PAP^65–79^, PAP^65–78^, PAP^66–79^, and PAP^66–78^ were observed, respectively, at 1610.78 [M + H]^+^, 1552.50, 1552.74 as shown in Supplementary Figure S1B, and 1517.951 [M + Na]^+^ (Supplementary Figure S1D).

### Preparation of PCIMPs

The synthesis of Fe_3_O_4_, Fe_3_O_4_@APTMS, Fe_3_O_4_@APTMS-GA, and Fe_3_O_4_@APTMS-GA-acrylate was carried out as described previously (Ding et al., 2006; Yang et al., 2017; Kanubaddi et al., 2021). Furthermore, for the preparation of PCIMPs using a cross-linker, 5.6 mM of the cross-linker (Metha-Asn-NHNH-Metha) and 0.014 mM of the template (PAP^65–79^, PAP^65-78^, or PAP^66-79^) were dissolved in 12 ml of trifluoroethanol (TFE)/H_2_O = 7: 3. For fabrication of PCIMPs using monomers, acrylamide (AA) (0.24 mM), *N*-Acr-L-His-NHBn (0.24 mM), *N*-acryltyramine (0.48 mM), and the cross-linker *N,N′*-ethylene bisacrylamide (0.84 mM), and 0.014 mM of the template (PAP^66-78^) were dissolved in 6 ml of TFE/ H_2_O = 7: 3. The combination was stirred for 1 h to make a pre-self-assembled mixture. Then 100 mg of Fe_3_O_4_@APTMS-GA-acrylate was added to the mixture and stirred for another 1 h, followed by 500 μl (10%, W/W) of ammonium persulfate (initiator) and 250 μl (5%, W/V) of tetramethylethylenediamine (TEMED), stirred at RT for 24 h in the presence of N_2_. Next, the resultant mixture was washed four times with 5% acetic acid (aq), containing 0.5% tween@20 and rinsed with H_2_O. Finally, the pore structures formed from different template molecules and were denoted as PCIMPs^65–79^, PCIMPs^65–78^, and PCIMPs^66–79^ (Kanubaddi et al., 2021). The compositions of other PCIMPs are given in Supplementary Table S1;these were synthesized using the same procedure.

### Physicochemical characterization of MPs and PCIMPs

The presence of PCIs fabricating on MPs was confirmed using Fourier transform infrared (FT-IR) spectroscopy (Bruker TENSOR 27, Ettlingen, Germany). The morphology of functionalized MPs and PCIMPs was observed under a Field Emission-Scanning Electron Microscope (FE-SEM, JOEL JSM-7000F/JEOL Ltd. Japan) equipped with an Oxford Instruments X-Max EDS system and operated at an acceleration voltage of 200 kV. The elemental analysis was performed by energy-dispersive X-ray spectroscopy (EDS). To demonstrate the amine groups on the surface of MPs, Ninhydrin reagent in the detection of grafted functional groups was assessed. For the Ninhydrin test (Albert Brown Ltd., Leicester, United Kingdom), the samples were placed into the Ninhydrin gel vials provided and incubated at 60°C for 30 min. The vials were then inspected and scored according to the following scale: 0, no color (negative); 1, slight purple color; and 2, dark purple color.

### Determination of binding affinities of PCIMPs

To prevent the non-specific binding sites' adsorption on PCIMPs, can binding experiments were carried out for a few minutes. Briefly, 20 mg of PCIMPs was suspended in 1 ml of H_2_O containing specific initial PAP concentrations (0.125, 0.25, 0.5, 1.0, and 2.0 mg/ml). After being shaken at 25°C for 5 min, the mixture was separated using a magnet. Then, 200 µl of the supernatant was taken out and measured using a Fluorescence Microplate Reader at *E*_ex_/*E*_em_ = 290 nm/350 nm. The binding affinities of PCIMPs were evaluated using the Scatchard analysis equation (1) (Gerdon et al., 2005; Diltemiz et al., 2009; Tai et al., 2012; Kanubaddi et al., 2021),

$$\left[\text{RL}\right]/\left[\text{L}\right]=\left({\text{B}}_{\max}-\left[\text{RL}\right]\right)/{\text{K}}_{\text{d}}$$ (1)

where [L] is the concentration of PAP in the solution, [RL] is the concentration of the bound PAP from the solution, *B*_max_ represents the maximum number of binding sites, and *K*_d_ is the ligand dissociation constant.

### Immobilization of papain (PAP/PCIMPs)

Briefly, 10 mg of PAP was dissolved in 1 ml of H_2_O and 20 mg of PCIMPs was added to the solution to incubate for 4 h. The resulting PAP/PCIMPs were collected and washed with H_2_O. Finally, the PAP/PCIMPs obtained were dried at 0°C and stored in a sealed vial at 4°C until further use.

### Determination of esterification activity of PAP and immobilization PAP (PAP/PCIMPs) by the ^1^H-NMR method

The esterification activity of PAP and PAP/PCIMPs was determined using the ^1^H-NMR method. The starting material was Boc-L-His-OH and the product was Boc-L-His-OMe, which was observed over time. The percentage of esterification rate was calculated by using the following equation (2):

$$Yield=\frac{Product\;integral\;values}{\left(Starting\;integral\;values+product\;integral\;values\right)}\times 100$$ (2)

To determine the esterification activity of PAP and PAP/PCIMPs, a solution of Boc-L-His-OH (0.1 M) was prepared in dry methanol (MeOH). Then, an enzymatic reaction in organic solvents was carried out, with modifications of earlier reported methods (Zaks and Klibanov, 1985; Belyaeva et al., 2002). First, 20 mg of PAP was added to Boc-L-His-OH (0.1 M) in 2 ml of MeOH, followed by 50 μl of water. Then, the reaction was carried out at 20°C for 48 h. For immobilized enzyme, 20 mg of PAP/PCIMPs was added to a Boc-L-His-OH (0.1 M) solution and incubated for 48 h, and the product concentration was monitored for 48 h with samples taken every 8 h by ^1^H-NMR. The same procedure was also used for the adsorption test. For de-adsorption, we used acetonitrilcan (ACN): H_2_O to remove the PAP, and repeated the process four times.

### Determination of kinetic constants of PAP and PAP/PCIMPs

The kinetic parameters of PAP and PAP/PCIMPs were determined using varying concentrations of Boc-L-His-OH (0.06, 0.08, and 0.1 M) in 2 ml of dry MeOH. Then, 20 mg of PAP/PCIMPs was added to each concentration and incubated. For every 8 h interval, 100 μl of the aliquot was withdrawn, dried in a vacuum, and monitored by ^1^H-NMR. Finally, the kinetic parameters of PAP and PAP/PCIMPs were evaluated using the Michaelis–Menten kinetics plot obtained from the following equation 3:

$$v=\frac{\left(V_{max}\left[\text{S}\right]\right)}{\left(\left(K_{m}\right)+\left[\text{S}\right]\right)}$$ (3)

where *v* is the velocity, *V*_max_ is the maximum rate of enzyme activity, [S] is the substrate’s concentration, and *K*_m_ is the Michaelis half-saturation constant.

The turnover number (*k*_cat_) was determined using the following equation 4:

$$k_{\text{cat}}=V_{\max}/\left[\text{E}\right]$$ (4)

where *V*_max_ is the maximum rate of enzyme activity and [E] is the concentration of the enzyme (Bossi et al., 2012).

### Reusability analysis

To demonstrate the reusability performance of the PCIMPs’ imprinted materials, stable catalytic activity was compared with the PAP-immobilized Sephadex G-25 (Tai et al., 1989) and PAP, respectively. The PAP/PCIMPs^65–79^ were also examined with the same catalytic activity at different times to determine reuse performance.

## Results and Discussion

### Rational selection of the template and its analysis

**FIGURE 2:**
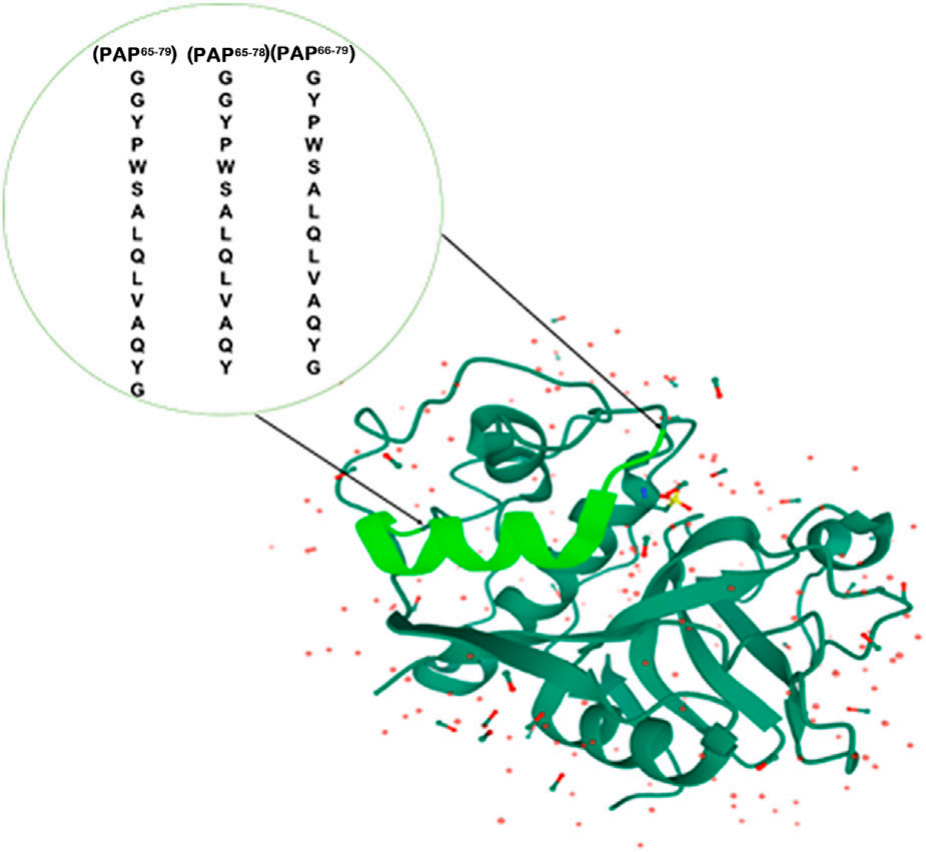
The selected sequence is in pale green. These segments consist of the series, i.e., PAP^65–79^, PAP^65–78^, and PAP^66–79^. (The crystal structure of PAP was reproduced from https://www.rcsb.org/3d-view/9PAP, and PDB ID: 9PAP.)

To obtain unique protein recognition on the surface of MPs, the helical peptide residues in the template are the critical parameters. The selection of template peptide fragments is based on the flank part of the spatial protein structure to limit their interference during catalysis. The length of the peptide segments such as 14–15**-**mer from the flexible structure on the surface of MPs can be helpful during the process of protein rebinding (Tai et al., 2011; Bossi et al., 2012; Kanubaddi et al., 2021). Therefore, the chosen 14–15**-**mer peptide sequences containing PAP^65–79^, PAP^65–78^, and PAP^66–79^ of the PAP were selected as a template. The location of the template is shown in Figure 2, and the list of the peptides is described in Table 1.

**TABLE 1:** List of the selected peptide segments as a template.

Sequence	Residue	Theoretical		MALDI-TOF MW (avg)
		pI^a^	MW (avg)^a^	
(PAP^65–79^) GGYPWSALQLVAQYG	15	5.52	1609.80	1610.78
(PAP^65-78^) GGYPWSALQLVAQY	14	5.52	1552.75	1552.50
(PAP^66-79^) GYPWSALQLVAQYG	14	5.52	1552.75	1552.74
(PAP^66-78^) GYPWSALQLVAQY	13	5.52	1495.70	1517.95

a pI and MW values were calculated from the Expasy website.

### Helical conformational analysis

**FIGURE 3:**
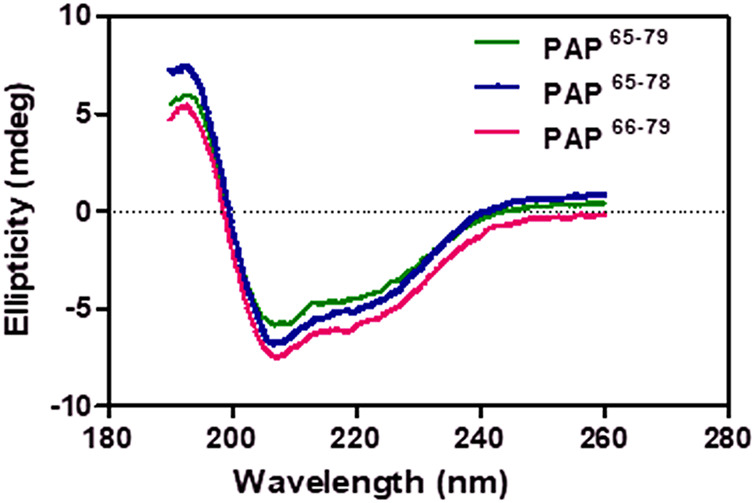
The CD spectra of PAP^65-79^, PAP^65–78^, and PAP^66–79^ segments.

The helical structure of our designed peptides (PAP^65–79^, PAP^65–78^, and PAP^66–79^) were examined using a J-715 spectropolarimeter (Jasco Inc., Japan). The synthesized peptides gave a well-defined secondary conformation by circular dichroism (CD) of the peptide solution (20 mM; TFE/H_2_O = 7:3). As shown in Figure 3, the CD spectra exhibited positive peaks at around 193 nm and two minimum negative bands at 207 and 218 nm, indicating α-helix structures’ predominance. Accordingly, the peptides were stabilized as a helical conformation in a mixed solvent (TFE/H_2_O) to allow the formation of helical cavities with a PCIMPs-based method (Chou et al., 2020; Kanubaddi et al., 2021).

### Preparation of a cross-linker (Metha-L-Asn-NHNH-Metha)

The synthesis of the cross-linker is straightforward, as outlined in Scheme 1. First, Boc-L-Asn-OH **1** was coupled with Boc-NHNH_2_ via EDC to generate **2**. The Boc protecting group of **2** was then deprotected using MeOH⋅HCl to obtain **3**. Finally, after acylating with twofold methacryloyl chloride, cross-linker **4** was obtained. The role of the synthesized cross-linker is also kind of a functional monomer. The cross-linker can form more rigid PCIMPs for improving the binding affinity and forming stable PCIMPs. It was attributed to cooperative hydrogen bonding or electrostatic interactions with the protein molecule. Moreover, it possesses an amino group that can easily interact with the substrate to enhance the productive catalytic activity of PAP/PCIMPs.

### Proposed mechanism of formation of PCIMPs and their interaction with the template and PAP

Because of self-assembly, the amino group of the cross-linkers attached to the template with ionic bonding and hydrogen bonding. Meanwhile, the monomers, cross-linkers, and Fe_3_O_4_@APTMSGA-acrylate were attached to each other with the hydrophobic acryloyl group. All the monomers and cross-linkers were thus attached to the template and Fe_3_O_4_@APTMS-GA-acrylate in a preorganized manner. Afterward, the formulation of PCIMPs took place in an organized manner. The free-radical polymerization process of a cross-linker was initiated by ammonium persulfate and TEMED. TEMED accelerates the rate of formation of free radicals from persulfate. These, in turn, catalyze polymerization. The persulfate free radical converts the methacrylate group of the cross-linker to free radicals, which react with unreacted ones to begin the polymer chain reaction. The elongating polymer chain is randomly cross-linked with Fe_3_O_4_@APTMS-GA-acrylate, resulting in the production of 3D polymer networks; the interaction of the polymer with the template via ionic bonding and hydrogen bonding to produce a template–PCIMPs complex. This is formed in a large excess of a crosslinking agent to form a 3D polymer network. After the polymerization process, template molecules are removed using an amphiphilic solvent. The role of the synthesized cross-linker is also of a dual-function monomer. The cross-linker can form more rigid PCIMPs to improve the binding affinity and stabilize PCIMPs. It was attributed to cooperative hydrogen bonding, ionic bonding, and electrostatic interactions with the protein molecule. Moreover, it possesses an amino group that can easily interact with the substrate to enhance the catalytic activity of PAP/PCIMPs.

### Physical characterization of MPs and PCIMPs

**FIGURE 4:**
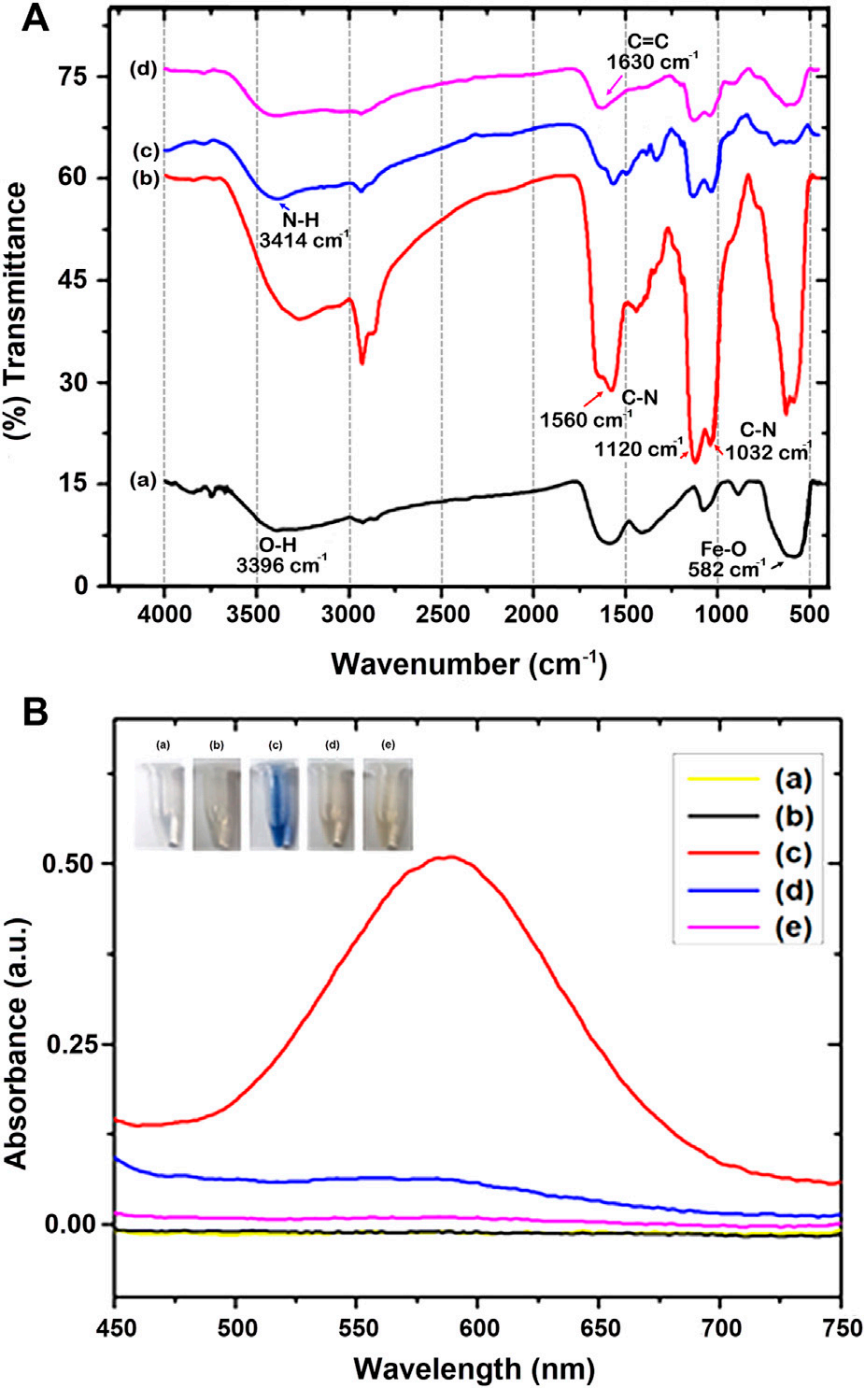
**(A)** FT-IR spectra of **(a)** Fe_3_O_4_, **(b)** Fe_3_O_4_@APTMS, **(c)** Fe_3_O_4_@APTMS-GA, and **(d)** Fe_3_O_4_@APTMS-GA-acrylate. **(B)** Ninhydrin assay **(a)** Ninhydrin, **(b)** Fe_3_O_4_, **(c)** Fe_3_O_4_-APTMS, **(d)** Fe_3_O_4_-APTMS@GA, and **(e)** Fe_3_O_4_-APTMS@GA-acrylate.

The fabrication of MPs is shown in Figure 4A, and is characterized by Fourier transform infrared (FT-IR) spectroscopy (Bruker TENSOR 27, Ettlingen, Germany). In the beginning, the bare Fe_3_O_4_ sample reflected only a few functional groups prominently, such as iron oxide and hydroxide groups. The broad peak at around 582 cm^−1^ was ascribed to the Fe-O, and a peak at 3,396 cm.^−1^ (Figure 4A,a). Later, APTMS immobilized on Fe_3_O_4_ resulted in additional peaks around 1,032 cm^−1^, 1,120 cm^−1^, and 1,560 cm.^1^ (C-N vibration), and a maximum at 3,420 cm^−1^, which could be ascribed to the silanol group (Si-O) and NH_2_ (Kurtan and Baykal, 2014; Farjadian et al., 2016; Yang Y. et al., 2018; Gao et al., 2018; Kanubaddi et al., 2021), respectively. These characteristic peaks of APTMS molecules indicate their successful coating on Fe_3_O_4_ (Figure 4A,b). In addition, the C-H stretching peaks at ∼ 2,900 cm^−1^ are due to the presence in the alkyl chain of APTMS_43-44_. Besides, no peak was observed at 1,739 cm^−1^, indicating that C=O at both ends of the glutaraldehyde functional groups reacted with NH_2_. This was attributed to the formation of a secondary amine. The unique peak at 3,414 cm^−1^ was ascribed to the N-H group (Figure 4A,c). Further, as seen in Figure 4A,d, the peak at 1,630 cm.^−1^, ascribed as the characteristic peak of C=C, suggests successfully conjugated acrylation on MPs (Secundo, 2013; Farjadian et al., 2015; Kanubaddi et al., 2021).

Furthermore, the successful modification of the amine group on the surface of Fe_3_O_4_ was confirmed by the Ninhydrin test (Figure 4B), using our established method (Kuo et al., 2015). We treated the modified Fe_3_O_4_ samples with a 1 ml Ninhydrin solution at 60°C for 30 min. After the reaction, the MPs were centrifuged. The solvent was measured in UV-Vis, and the characteristic absorption of Ruhemann’s purple was shown at 580 nm., which can be labeled as primary amine-modified on the surface of Fe_3_O_4_. However, when treated with secondary amine-modified Fe_3_O_4_ nanoparticles (Fe_3_O_4_@APTMS-GA), ninhydrin cannot produce Ruhemann’s purple color (Kuo et al., 2015). When we compared Ruhemann’s purple absorbent intensities, Fe_3_O_4_-APTMS showed high intensity, whereas Fe_3_O_4_@APTMS-GA and Fe_3_O_4_@APTMS-GA-acrylate samples showed low intensities. This shows that GA is successfully modified on the amine-modified Fe_3_O_4_ nanoparticles. The reactivity of ninhydrin with various surface-functionalized Fe_3_O_4_ samples provides further evidence to confirm successive chemical modifications on the Fe_3_O_4_ surfaces.

**FIGURE 5:**
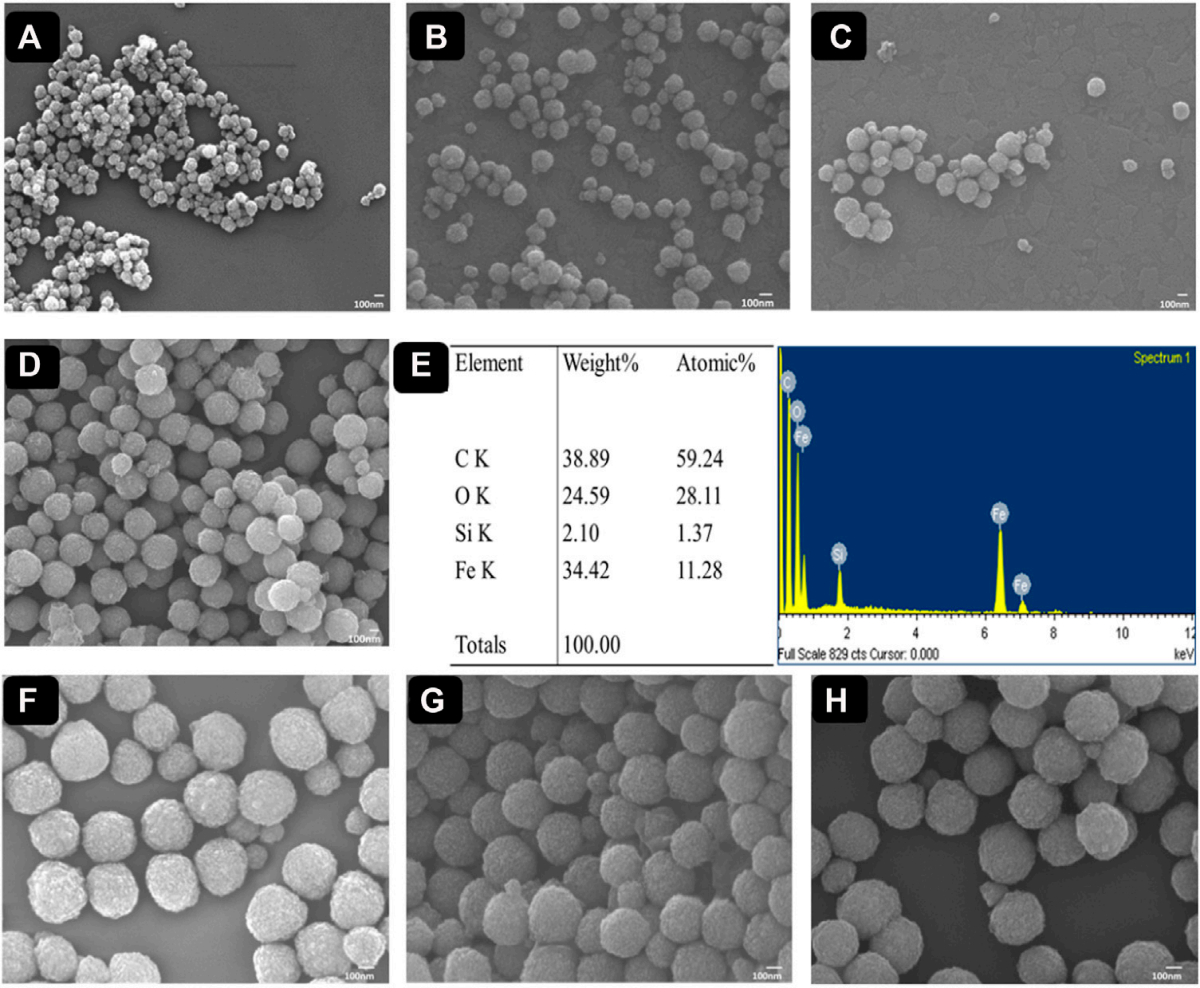
FE-SEM images of **(A)** Fe_3_O_4_, **(B)** Fe_3_O_4_@APTMS, **(C)** Fe_3_O_4_@APTMS-GA, and **(D)** Fe_3_O_4_@APTMS-GA-acrylate. The EDX spectrum image of **(E)** Fe_3_O_4_@APTMS-GA-acrylate. FE-SEM images of **(F)** PCIMPs^65–79^, **(G)** PCIMPs^65–78^, and **(H)** PCIMPs^66–79^.

Eventually, the surface morphology of functionalized MPs and PCIMPs was subsequently characterized by Field Emission-Scanning Electron Microscopy (FE-SEM) observations (JEOL JSM-7000F/JEOL Ltd. Microscope from Tokyo, Japan). It was apparent from the FE-SEM images of Fe_3_O_4_ nanoparticles that their successively surface-functionalized samples were uniform, and their spherical shape can be seen in Figure 5. We determined the shape of MPs and PCIMPs based on the Feret diameter analysis measurement. The diameter of each particle was calculated on one axis of the particle. We also measured the size of the particle directly based on its FE-SEM image. The FE-SEM image shows that Fe_3_O_4_ is a spherical shape, with an average size of ∼142 nm, as shown in Figure 5A. Moreover, APTMS coated on the surface of Fe_3_O_4_. An increase in particle size was observed, the average size being ∼175 nm, indicating the successful immobilization of APTMS on Fe_3_O_4_, (Figure 5B). Further, immobilization of GA on modified MPs resulted in a uniform shape and size of ∼228 nm (Figure 5C). Moreover, acrylate functionalization on modified MPs shows that the average size was ∼260 nm (Figure 5D). The elemental analysis from the Energy-dispersive X-ray Spectroscopy (EDS) image (Figure 5E) showed that Fe_3_O_4_@APTMS-GA-acrylate possessed the highest content of the C element over Fe, Si, O, and N species. Successful modification of acrylate on modified MPs was also observed. Among all PCIMPs, as shown in Figures 5F–H, the 15-mer fabricated PCIMPs^65–79^ were comparatively larger, around 375 nm. Other 14-mer fabricated PCIMPs^65-78^ and PCIMPs^66-79^ showed similar sizes (∼360 nm.).

### Fabrication of PCIMPs to capture PAP

To further improve the PCIMPs’ fabrication, it was also necessary to create recognition sites that are complementary to protein recognition. The L-asparagine derivative (Metha-Asn-NHNH-Metha) was introduced as a cross-linker. The role of the cross-linker is to control the surface morphology of the polymer matrix and stabilize the imprinted binding sites, retaining their molecular recognition capability (Sellergren, 1999; Vasapollo et al., 2011). As shown in Supplementary Table S1, PCIMPs were fabricated with different concentrations of cross-linker and template. As the amount of the cross-linker and template increased, it helped to increase the binding affinity toward the mother protein. Meanwhile, adding a cross-linker formed more rigid PCIMPs and resulted in a more stable catalyst.

The PCIMPs-grafted 15- and 14-mer peptides were then tested for their ability to bind their mother protein, as described previously (Tai et al., 2012). As shown in Table 2, it was found that PCIMPs^65–79^ had more significant binding affinities, while the other two, PCIMPs^65-78^ and PCIMPs^66-79^, exhibited lower affinity. It was previously reported that the higher the number of residues in the template, the better binding affinity was observed (Tai et al., 2011; Kanubaddi et al., 2021). Accordingly, for PCIMP-grafted 15-mer peptides, the best *K*_d_ value was 0.087 μM, and it had a better affinity than the PCIMP-griated 14-mer peptides. Interestingly, among PCIMP-grafted 14-mer peptides, PCIMPs^66-79^ had the best *K*_d_ value (0.13 μM), whereas PCIMPs^65-78^ exhibited the lowest value (0.17 μM). Moreover, our developed PCIMPs generated binding sites that complemented protein recognition and consequently produced higher affinity toward targeted protein PAP. The elegant helical cavities stamping approach was compared with the other MIPs-grafted methods, based on the binding affinities and absorption time. Our results showed a higher affinity of PAP to PCIMPs^65–79^ than other observed MIPs-grafting approaches (Yang et al., 2016; Xu et al., 2018; Boitard et al., 2019).

**TABLE 2:** Optimization of the formation of PCIMPs and affinity values toward PAP.

Formulation	Residue	Template (mM)	Monomers (mM)	Cross-linker (mM)	*K*_d_ (μM)	*B*_max_ (μM)	Yield (%)
PCIMPs^65–79^	15	PAP^65–79^ (∼0.014)	—	5.6^a^	0.087	4.56	89
PCIMPs^65–78^	14	PAP^65–78^ (∼0.014)	—	5.6^a^	0.17	0.52	77
PCIMPs^66–79^	14	PAP^66–79^ (∼0.014)	—	5.6^a^	0.13	0.49	79.2
PCIMPs^66–78^	13	PAP^66–78^ (∼0.014)	AA (0.24), Acr-L-His-NHBn (0.24), N-acryl tyramine (0.48)	0.84^b^	0.097	3.45	84.5
NIP	—	—	—	—	—-	—-	<5

Note: 100 mg of Fe_3_O_4_@APTMS-GA-acrylate was used in the preparation of PCIMPs. The volume of all polymerization solvents was 12 ml (TFE/H_2_O = 7: 3). Monomers (AA, acrylamide, Acr-L-His-NHBn, and *N*-acryl tyramine), cross-linkers = ^a^Metha-Asn-NHNH-Metha and ^b^EBAA (*N,N*′-ethylene bisacrylamide). NIP, non-imprinted polymer. The purity of the template plays an important role in the binding affinity. For example, 13**-**mer peptide has a high purity of ∼95% compared with the 14**-**mer (∼88%). Therefore, 13**-**mer grafted PCIMPs^66-78^ have higher binding affinity when compared to those of 14**-**mer grafted PCIMPs^66-79^ and PCIMPs^65-78^.

The traditional synthetic method developed for 13**-**mer grafted PCIMPs^66-78^ was also compared with the fabricated PCIMPs with the cross-linker to detect PAP. In this preparation method, to improve the fabrication of PCIMPs^66-78^, we introduced two monomers, chiral histidine derivative (Acr-L-His-NHBn) and *N*-acryl tyramine. Interestingly, as displayed in Table 2, we observed that 13**-**mer-grafted PCIMPs^66-78^ led to higher affinity toward the analyte (*K*_d_ = 0.097 μM) when compared to fabricated 14**-**mer-grafted PCIMPs using the cross-linker (Metha-Asn-NHNH-Metha), i.e., PCIMPs^65-78^ (*K*_d_ = 0.17 μM) and PCIMPs^66-79^ (*K*_d_ = 0.13 μM). Moreover, the traditional formulated 13**-**mer-grafted PCIMPs had a better affinity toward PAP than the 14**-**mer-grafted PCIMPs using the cross-linker (Tai et al., 2011). Furthermore, monomers such as Acr-L-His-NHBn and *N*-acryl tyramine helped to strengthen affinity for PAP and helped harden the surface of the polymer matrix on PCIMPs,^66-78^ and reduced swelling while imprinting integrity (Yang et al., 2013). In addition, adding the cross-linker (EBBA) formed more rigid PCIMPs^66-78^ and produced a more stable catalytic activity (Tai et al., 2011). There was also a tendency for longer peptide residues to sustain a stable conformation throughout the polymerization process. For instance, 15 mer-grafted PCIMPs^65–79^ fabricated with Metha-Asn-NHNH-Metha showed a higher binding affinity toward PAP compared to the 13**-**mer and 14**-**mer PCIMPs. Therefore, fabricated PCIMPs^65–79^ using a cross-linker resulted in better protein binding and higher catalytic performance.

### Evaluation of esterification activity of PAP and PAP/PCIMPs by using ^1^H-NMR

**FIGURE 6:**
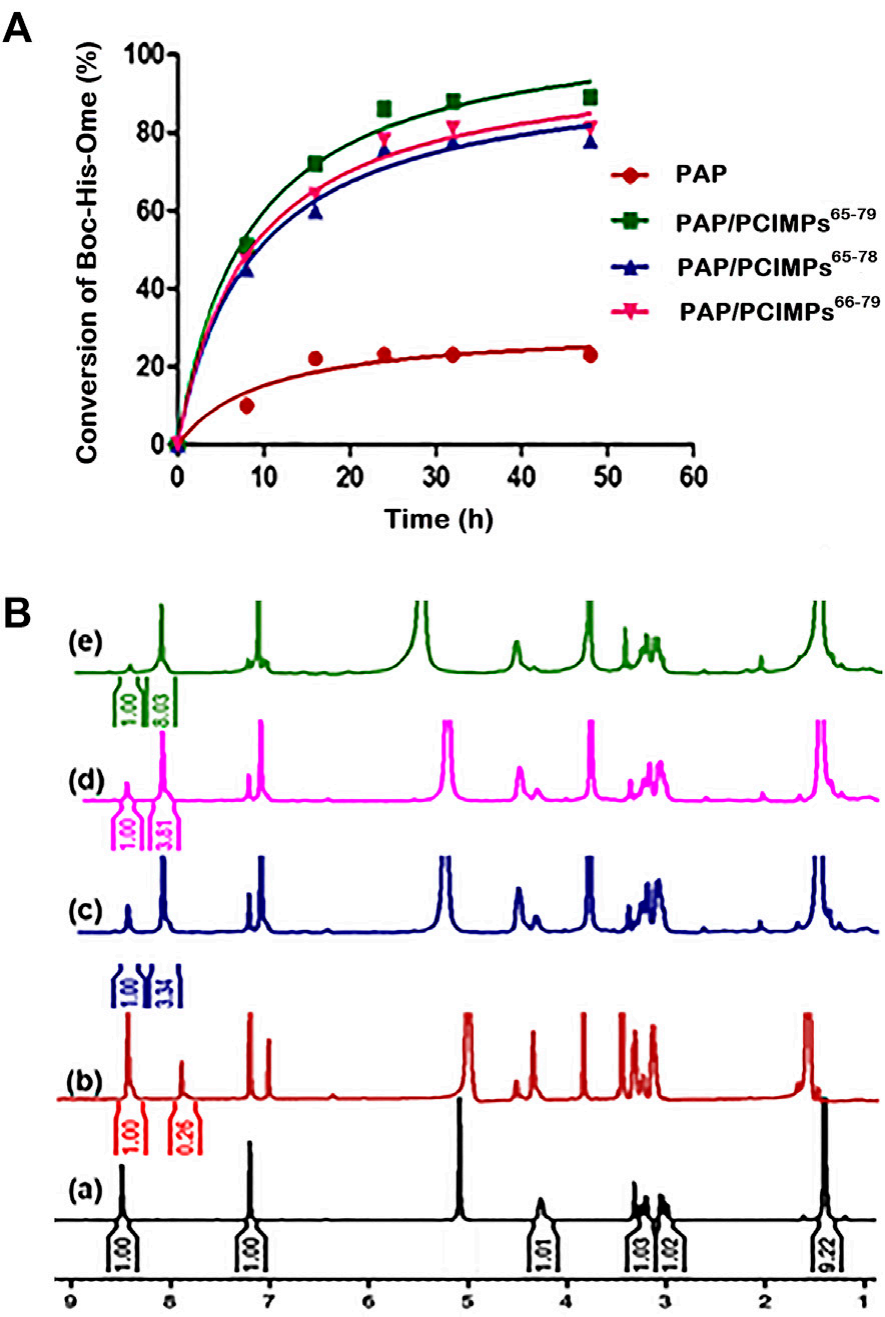
**(A)** Results of esterification of Boc-His-OH at 35°C at various intervals of reaction times. **(B)** ^1^H-NMR spectra of **(a)** the starting material (Boc-His-OH), and the reaction mixture of the starting material and ester formed by **(b)** PAP, **(c)** PAP/PCIMPs^65–78^, **(d)** PAP/PCIMPs^66–79^, and **(e)** PAP/PCIMPs^65–79^ (no of scans used = 32, solvent = CD_3_OD).

The maximum conversion of our PAP/PCIMPs was achieved in 24 hours (Figure 6A). Thus, the same duration (i.e., 24 hours) was chosen for the study of PAP/PCIMPs. The performance of the fabricated PAP/PCIMPs^65–79^ in catalyzing the esterification of *N*-protected amino acid was compared with the previous results. Accordingly, PAP/celite catalyzed esterification of *N*-protected amino acids in various organic solvents for 4 days. The desired product was obtained by silica gel chromatography, and purified esters were measured with ^1^H NMR. Their highest conversion was 71%, and reusability was not measured in this study (Shih et al., 1997). In another study, PAP was immobilized separately with eight kinds of adsorbents in a buffer solution. Among them, Sephadex G-50 was found to be the best adsorbent for immobilization of PAP. Accordingly, the PAP/Sephadex G-50 catalyzed esterification of *N*-substituted amino acids in MeOH for 2 days. Although this method shows a significant yield in esterification compared with other studies, PAP/Sephadex G-50 was difficult to reuse (Tai et al., 1989). Upon a comprehensive evaluation of *N*-protected amino acid esterification, the PCIMPs approach provided significant advantages over other methods. It was evident that the helical cavities created recognition sites on the MP surface and tightly bound the enzyme. As a result, it demonstrated better catalytic activity for esterification in comparison with previously conducted studies. The best catalytic performance of PAP/PCIMPs^65–79^ for the esterification of Boc-L-His-OH was 89% in 24 h.

The data suggested a trend that initially crude PAP catalyzed hydrolysis of substrates rapidly. However, due to the amount of substrate decreasing, the rate of hydrolysis gradually decreased after 8 h; the reaction reached equilibrium after 48 h. The PAP reaction was found to produce the lowest final yield (20.6%), which can be attributed to certain protein instability features in organic solvents, thereby decreasing enzyme activity (Stepankova et al., 2013). As for PAP/PCIMPs, the highest yield (89%) was attained with PAP/PCIMPs^65–79^ in 24 h. The performances of PAP/PCIMPs^66-79^ and PAP/PCIMPs^65-78^ were lower, at 79.2% and ∼77%, respectively. This shows that PCIMPs’ imprinted materials are highly stable in organic solvents when compared to crude PAP, and enhance the yield of esterification for up to 48 h. In comparison, the yield catalyzed by traditional formulated PAP/PCIMPs^66-78^ was 84.5% (Supplementary Figure S3), higher than those of PAP/PCIMPs^66-79^ and PAP/PCIMPs^65-78^, but 4% lower than that of PAP/PCIMPs^65–79^.

To measure the reaction yield accurately, esterification yields were characterized using ^1^H-NMR. The spectra were acquired at a frequency of 300 MHz on a Bruker (from Billerica, Massachusetts, United States) and Ultrashield (9.4 T) spectrometer using a 5-mm BBo probe at 296.2 K. To calculate the conversion of Boc-L-His-OH to Boc-L-His-OMe, the ratio between the integral relevant to the hydrogen group of the imidazole ring of Boc-L-His-OH and corresponding to the hydrogen group of Boc-L-His-OMe was compared to calculate the yield of the reaction based on Equation 2. As shown in Figure 6B,a, the chemical displacement at 8.5 ppm was a hydrogen group of the imidazole of the original material (Boc-L-His-OH), while the proton value was equal to 1. The imidazole of the product (Boc-L-His-OMe) was similar, at 8 ppm. The finding of a peak at 3.8 ppm was representative of α proton.

### Kinetic parameters of PAP and PAP/PCIMPs

**FIGURE 7:**
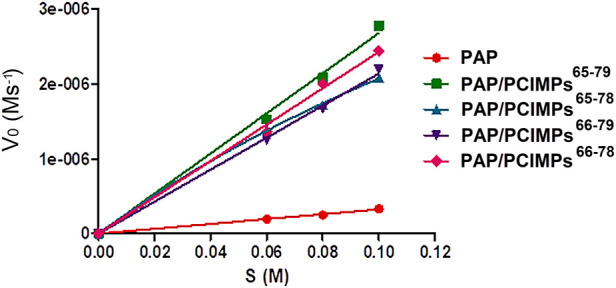
Michaelis–Menten plot of PAP and PAP/PCIMPs obtained with various concentrations of Boc-His-OH.

The Michaelis–Menten equation was applied to determine the enzyme kinetic parameters for PAP catalyzed reaction. In the present study, kinetic parameters of PAP and PAP/PCIMPs were determined by changing the Boc-L-His-OH concentration from 0.06 to 0.1 M. It was then calculated using the Michaelis–Menten plot, as shown in Figure 7.

As shown in Table 3, the *V*_max_ values were found to be 0.33 and 3.05 μM s^−1^ for PAP and PAP/PCIMPs^65–79^, while the performances of PAP/PCIMPs^65-78^ and PAP/PCIMPs^66–79^ were at 2.08 μM s^−1^ and 2.2 μM s^−1^, respectively. The *K*_m_ of PAP was found to be the highest at 5.5 × 10^−2^ M. The lowest *K*_m_ value was attained with PAP/PCIMPs^65–79^ as 5 × 10^−2^ M, whereas the *K*_m_ value for PAP/PCIMPs^65-78^ and PAP/PCIMPs^66–79^ were 5.2 × 10^−2^ and 5.3 × 10^−2^ M, respectively. The *K*_m_ value in PAP/PCIMPs was lower than that in the native enzyme. Consequently, PAP/PCIMPs have a greater affinity for the substrate than the PAP (Chen et al., 1998; Meridor and Gedanken, 2013). According to our calculation, PAP/PCIMPs possess the higher *V*_max_ value and the lower *K*_m_ value. Among all PAP/PCIMPs, PAP/PCIMPs^65–79^ have the best kinetic parameters to effectively promote the activity of PAP after immobilization (Sun et al., 2018).

**TABLE 3:** Kinetic parameters obtained from the Michaelis–Menten kinetics plot.

	PAP	PAP/PCIMPs^65-79^	PAP/PCIMPs^65-78^	PAP/PCIMPs^66-79^	PAP/PCIMPs^66-78^
*V*_max_ (M⋅s^-1^)	3.3 × 10^−7^	3.05 × 10^−6^	2.08 × 10^−6^	2.2 × 10^−6^	2.4 × 10^−6^
*K*_m_ (M)	5.5 × 10^−2^	5 × 10^−2^	5.2 × 10^−2^	5.3 × 10^−2^	5.3 × 10^−2^
*k*_cat_ (s^−1^)	7.8 × 10^−4^	**1.1 × 10** ^**−1**^	6.8 × 10^−3^	7 × 10^−2^	7.2 × 10^−2^
*k*_cat_/*K*_m_ (M^−1^⋅s^−1^)	0.014	2	1.3	1.32	1.35

Note: 13**-**mer grafted PCIMPs^66–78^ were prepared using Acr-L-His-NHBn and *N*-acryl tyramine monomers to help harden the polymer matrix’s surface and form a strong affinity for the PAP compared to the 14**-**mer-grafted PCIMPs. Furthermore, adding a cross-linker (EBBA) developed more rigid PCIMPs^66–78^ and produced a more stable catalytic activity. Therefore, PCIMPs^66–78^ contain better esterification kinetic values than those of PCIMPs^66–79^ and PCIMPs^65–78^. PAP, papain; PCIs, peptide conformational imprints; PCIMPs, peptide conformational imprint magnetic particles; PCIMPs66-78, PCIMPs-grafted 13-mer peptide; PCIMPs65-78 and PCIMPs66-79, PCIMPs-grafted 14-mer peptides; PCIMPs65-79, PCIMPs-grafted 15-mer peptide.

As shown in Table 3, the turnover (*k*_cat_) value of PAP (7.8 × 10^−4^ s^−1^) was lower than those of PAP/PCIMPs^65–79^ (1.1 × 10^−1^ s^−1^), PAP/PCIMPs^65-78^ (6.8 × 10^−2^ s^−1^), and PAP/PCIMPs^66–79^ (7 × 10^−2^ s^−1^), which could be attributed to the higher catalytic efficiency of immobilized PAP than the free enzyme. The higher *k*_cat_ value was possible due to the favorable interaction between the carrier (PCIMPs) and the enzyme (PAP), which addressed the PAP fold into the optimized conformation on the PCIMPs’ surface. Among all PAP/PCIMPs, the highest *k*_cat_ value for PAP/PCIMPs^65-79^ was 1.1 × 10^−1^ s^−1.^^.^, and were 6.8 × 10^−2^, and 7 × 10^−2^ s^−1^ or PAP/PCIMPs^65-78.^ and PAP/PCIMPs^66-79^, respectively. Similarly, the catalytic efficiency (*k*_cat_/*K*_m_) value of the PAP was 0.014 M^−1^ s^−1^, whereas it was 2 M^−1^ s^−1^ for PAP/PCIMPs^65–79^;PAP/PCIMPs^65–78^ and PAP/PCIMPs^66-79^ had similar values (∼1.3 M^−1^ s^−1^). The values of all PAP/PCIMPs were higher than that of the free PAP. This is probably because PAP/PCIMPs can maintain enzymatic activity and preserve favorable enzyme conformations (Wong et al., 2017; Yang L. et al., 2018; Li et al., 2019).

Moreover, we examined the kinetic parameters of traditionally formulated PAP/PCIMPs^66-78^ by increasing the Boc-L-His-OH concentration from 0.6, 0.8, and 0.1 M to 0.66, 0.88, and 0.11 M. As shown in Table 3, the *V*_max_ value of PAP/PCIMPs^66-78^ was 2.4 μM s^−1^, which was higher than those of PAP/PCIMPs^65-78^ (2.08 μM s^−1^), and PAP/PCIMPs^66-79^ (2.2 μM s^−1^) fabricated with Metha-Asn-NHNH-Metha. Thus, evidence supporting higher substrate concentrations increased considerably, while reaction rate increased significantly, changing the kinetic parameters. Thus, we can conclude that it appears worthwhile to increase substrates for PAP/PCIMPs^66–78^.

### Reusability

Another promising feature of PCIMPs is the rebinding of proteins and reusability. Once the PCIMPs are used for the application, the imprinted material used should be desorpted and reused. This will result in the sustainable repeated use of the material.

**FIGURE 8:**
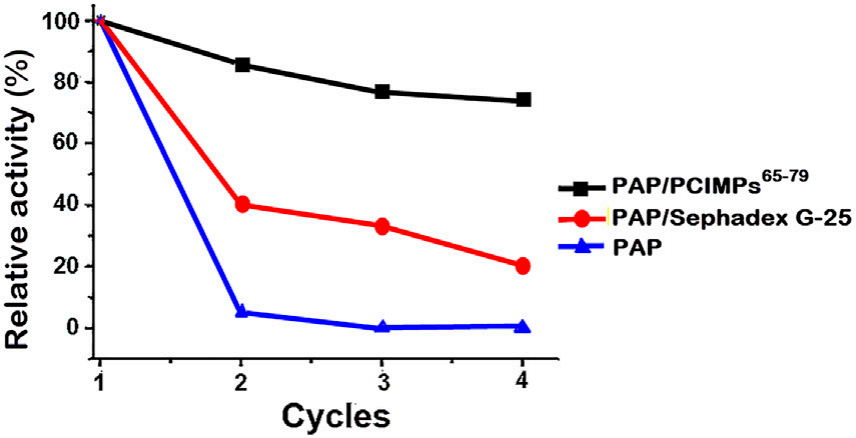
Reusability of PAP/PCIMPs^65–79^, PAP/Sephadex-G-25, and PAP.

To demonstrate regeneration ability and reusability of imprinted materials of PCIMPs^65–79^, adsorption–desorption cycles were repeated four times using the same imprinted material, as shown in Figure 8. The partially dried imprinted materials of PCIMPs containing methanol were blow-dried in a fume hood, and the damp PCIMPs-imprinted material was used for subsequent runs. The resulting product was preserved at 0°C for future use.

The amount of Boc-L-His-OMe-produced yield in the first cycle was set as 100%. The yield of Boc-L-His-OMe slightly declined after the first reuse. However, the yield remained around 80% in the last two cycles, proving that imprinted polymer particles can be regenerated and reused. Additionally, PAP/PCIMPs exhibited stable catalytic activity in the reusability studies, compared with the PAP/Sephadex G-25 and PAP. PAP/Sephadex G-25 declined to a 20% yield after the fourth cycle, and PAP did not show any significant reusability. Long-term stability and reusability of PAP/PCIMPs were high compared to the PAP/Sephadex G-25 and PAP.

## Conclusion

In summary, we succeeded in synthesizing a new chiral cross-linker, Metha-Asn-NHNH-Metha, from L-asparagine. Fabrication of the helical peptides using this cross-linker to form PCIMPs was accomplished by molecular imprinting technology. Among all the helical peptides used, 15-mer PCIMPs^65–79^ gained the highest binding affinity toward PAP and achieved the highest catalytic activity. Therefore, the fabrication of PCIMPs can provide an easy route to develop specific protein binding/adsorption and maintain enzyme catalytic activity.

Although trypsin flexibility interfered with the inhibitive effect on capturing the α-helix region during hydrolysis (Kanubaddi et al., 2021), PCIMPs were found to improve papain stability and robustness for esterification. This could be because the amount of water in esterification is much reduced in the reaction medium resulting in greater stability/activity of PAP/PCIMPs than PAP itself. The reuse displayed a small decrease in catalytic activity after four consecutive usages of the immobilized enzyme. Therefore, we conclude that our investigations will lead to more applications of these types of nanobiocatalysts in the future.

## Data Availability

The original contributions presented in the study are included in the article/Supplementary Material. Further inquiries can be directed to the corresponding authors.
