# Supplementary material for: Peptide conformational imprints enhanced the catalytic activity of papain for esterification
Source: Front Bioeng Biotechnol. 2022 Aug 16;10:943751. doi: 10.3389/fbioe.2022.943751 (PMC9424681; doi:10.3389/fbioe.2022.943751)
Supplement: Supplementary file 1 [file DataSheet1.docx]

Supplementary Material

1. **Experimental Section**
   1. **Materials**

3***-*(**aminopropyl**)** trimethoxysilane **(**APTMS**)**, and ammonium acetate **(**NH4Ac**)** from Acros Ltd (Fair Lawn, New Jersey, United States). *N*-benzyl acrylamide (BAA), and *N*,*N*-Ethylene bisacrylamide (EBAA) were acquired from Lancaster (Lancashire, UK). Iron **(**III**)** chloride hexahydrate **(**FeCl3***.***6 H2O**)**, and triethylamine **(**TEA**)** were purchased from Merck (Kenilworth, New Jersey, United States). Glutaraldehyde **(**GA**)** from Ferax (Berlin, Germany), all Fmoc amino acids obtained from BAChem (Bubendorf, Switzerland)***.*** Acetic acid, Acrylamide (AA), Boc***-***L***-***asparagine **(**Boc***-***Asn***-***OH**)**, Boc-hydrazide (Boc-NHNH2), sodium cyanoborohydride **(**NaBH3CN**)**, papain (PAP), tween®20, urea were purchased from Sigma***-***Aldrich (St. Louis, MO, United States)***.*** Acetone, acetonitrile **(**ACN**)**, dichloromethane **(**DCM**)**. *N*,*N****-***dimethyl formamide **(**DMF**)**, piperidine, toluene was HPLC grade***.*** Milli***-***Q, water purification system, purified distilled water used in all experiments***.***

1. **Analysis of a Template**

**Figure S1.** (a) HPLC chromatograms of PAP ^65-79^, PAP ^65-78^, and PAP ^66-79^. (b) MALDI spectrums of PAP ^65-79^, PAP ^65-78^, and PAP ^66-79^. The signal for intact PAP ^65-79^ found at m/z = 1610.78 (calculate mass = 1609.78), PAP ^65-78^ found at m/z = 1552.50 (calculated mass = 1552.73), and PAP ^66-79^ found at m/z = 1552.74 (calculate mass = 1552.73).. (c) HPLC chromatogram of PAP66-78. (d) MALDI spectrum of PAP66-78 founded at m/z = 1517.951[M+Na]+ (calculate mass = 1495.758).

1. **Characterizations of the Cross-linker (Metha-Asn-NHNH-Metha)**

**Complete compound 2 characterization data**

**
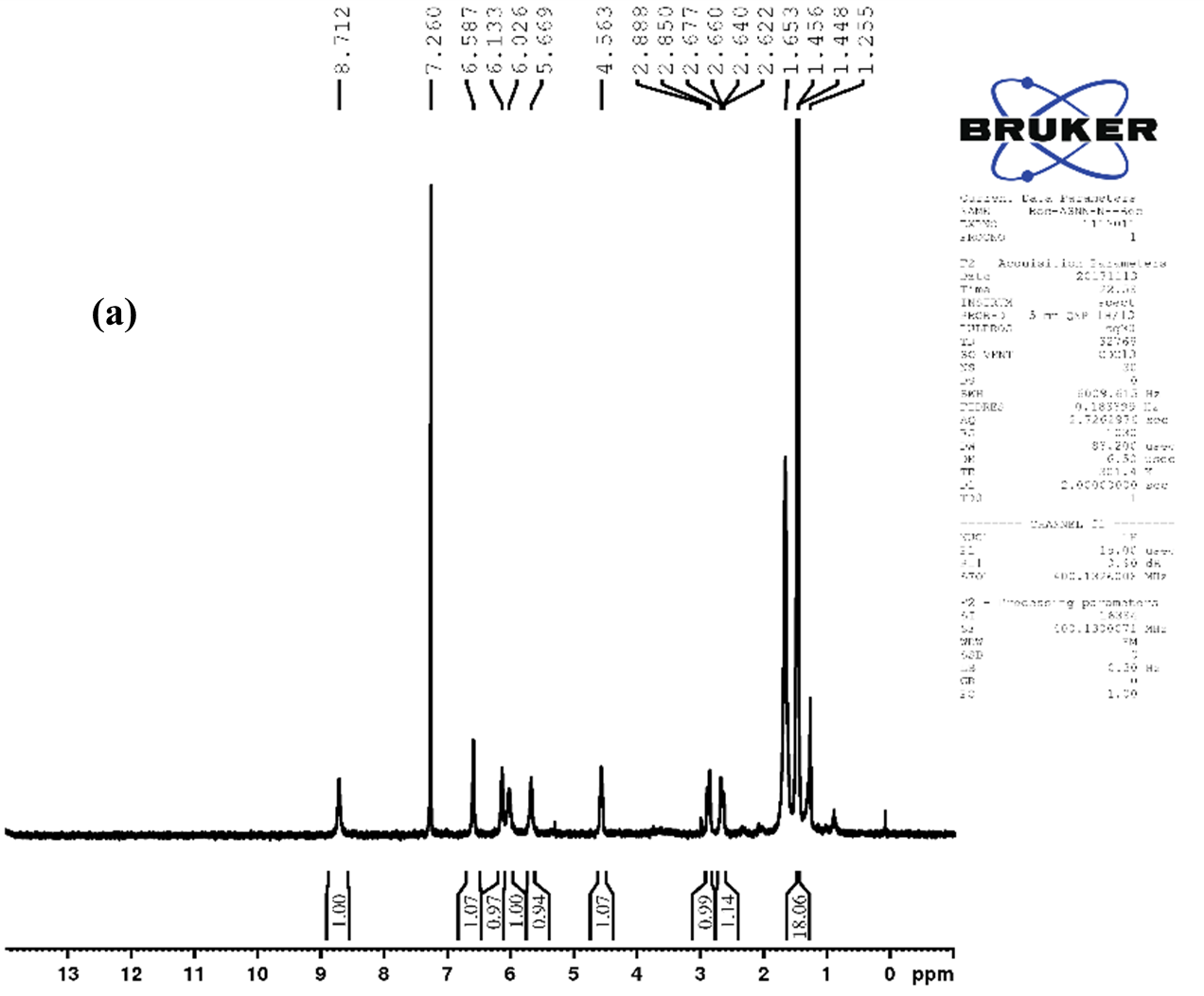
**

**Figure S2a.** The ^1^H-NMR spectrum of Boc-Asn-NHNH-Boc

**
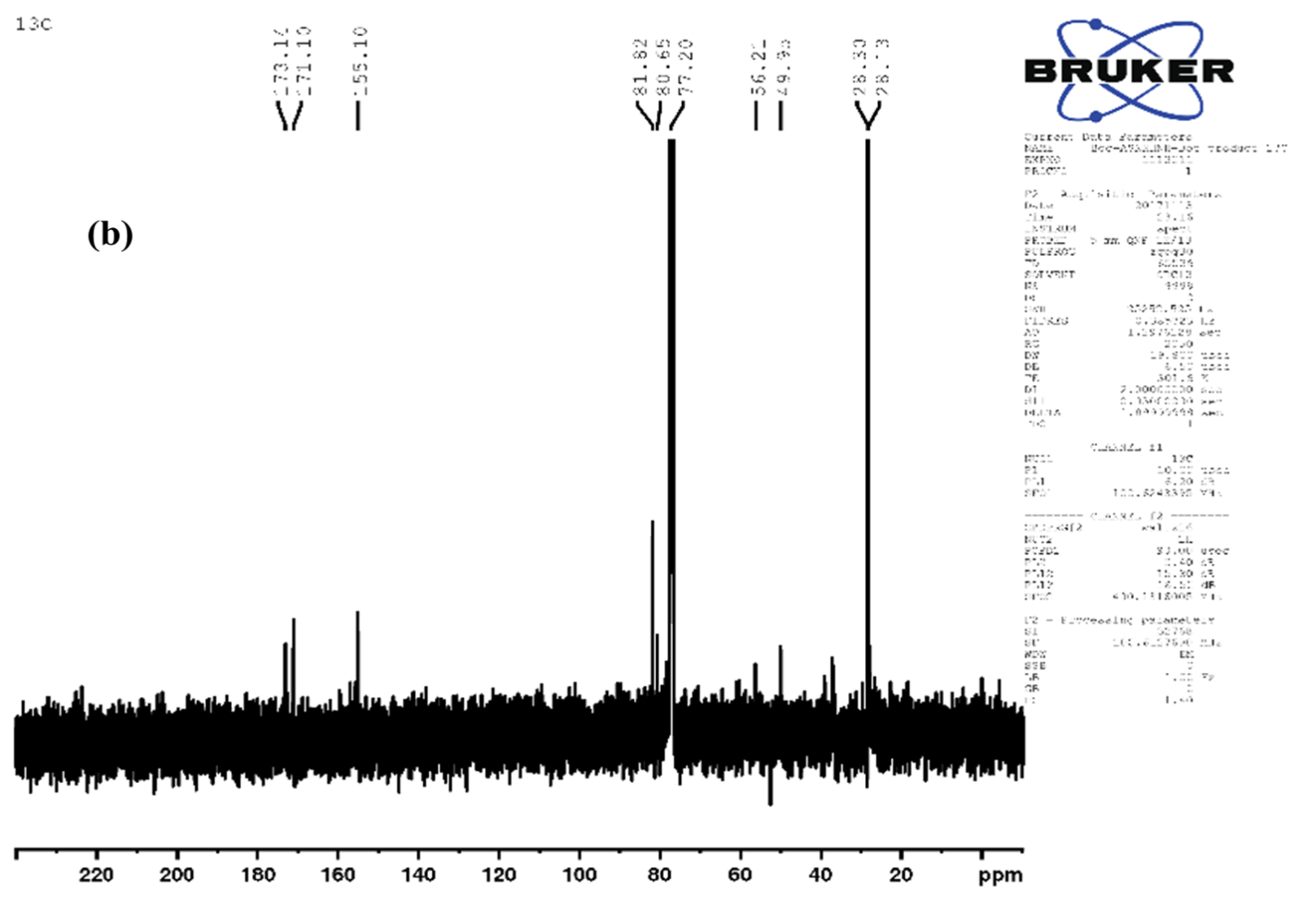
**

**Figure S2b.** The ^13^C-NMR spectrum of Boc-Asn-NHNH-Boc

**
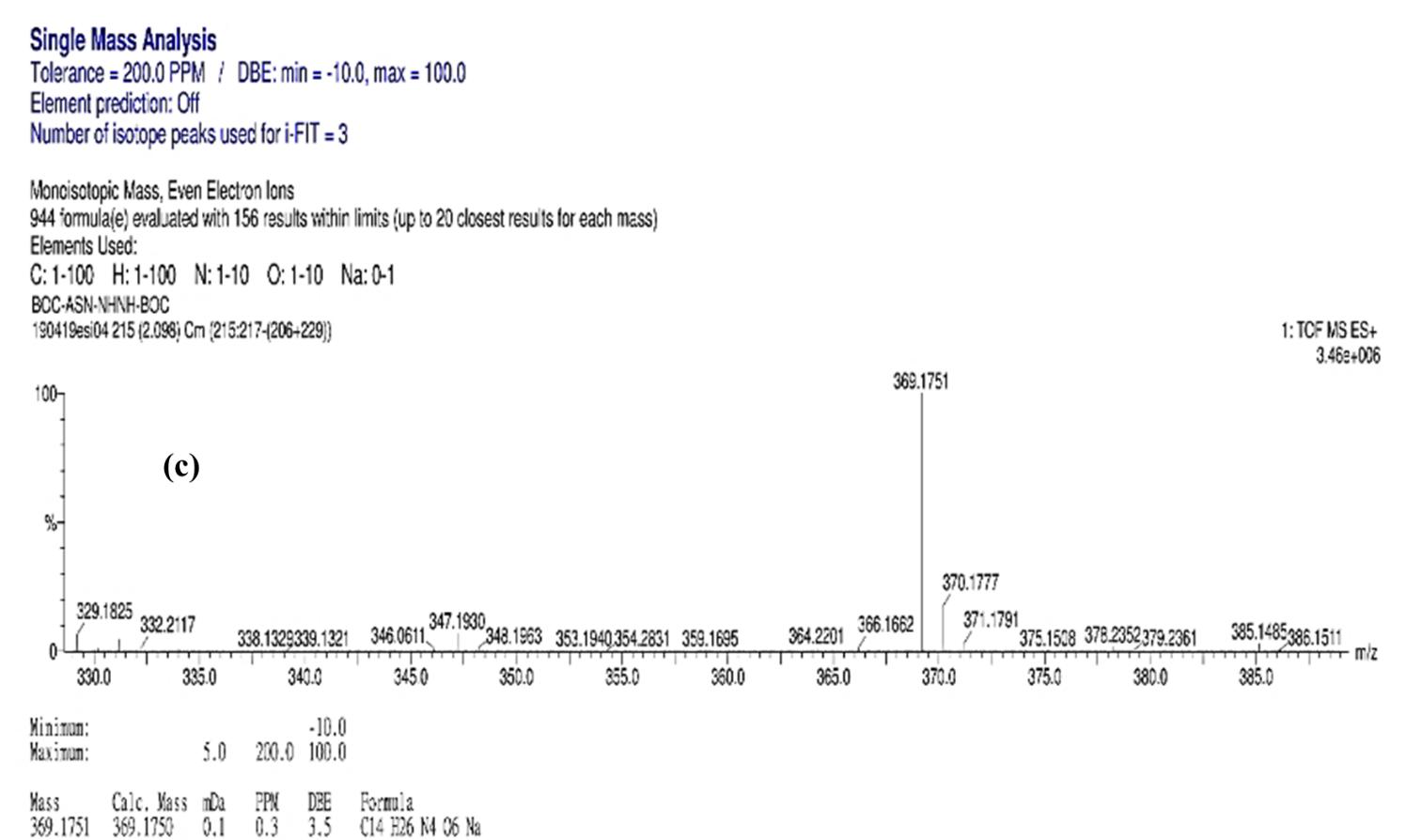
**

**Figure S2c.** The Mass spectrum of Boc-Asn-NHNH-Boc

**Complete compound 3 characterization data**

**
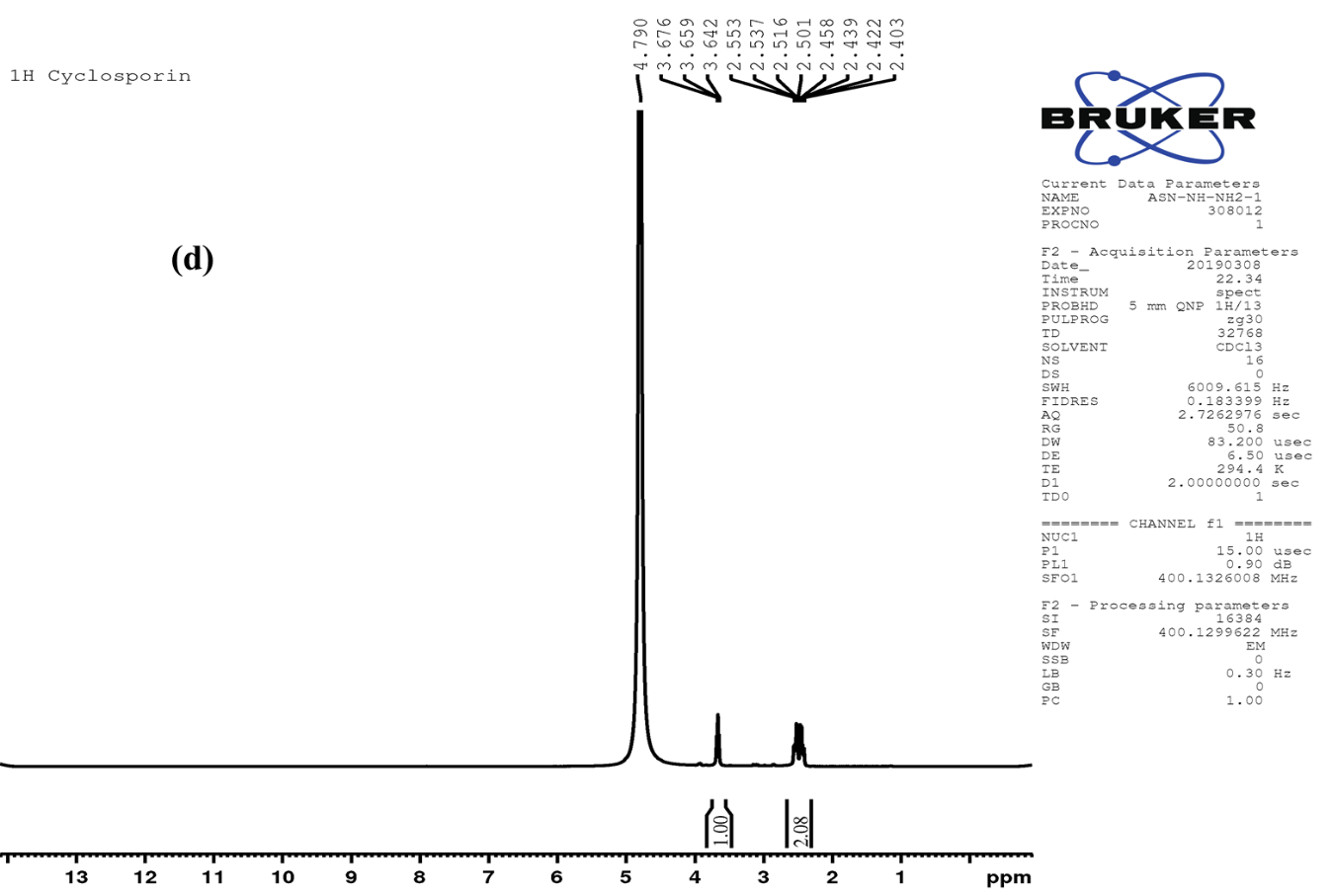
**

**Figure S2d.** The ^1^H-NMR spectrum of Asn-NHNH_2_.

^1^H-NMR (400 MHz, CDCl_3_-d_6_) δ (ppm): 8.7 (s, 1H), 6.5 (s, 1H), 6.1-6.0 (d, 2H), 5.6 (s, 1H), 4.5 (s, 1H), 2.8 (d, 1H), 2.6 (d, 1H), 1.4 (s, 18H). ^13^C-NMR (400 MHz, CDCl_3_-d_6_) δ (ppm): 173.1, 171.1, 155.1, 81.8, 80.6, 77.2, 56.2, 49.5, 28.3, and 28.1. HRMS (ESI): m/z calcd. For C_14_H_26_N_4_O_6_ [M+Na]^+^ 369.1750; found 369.1751.


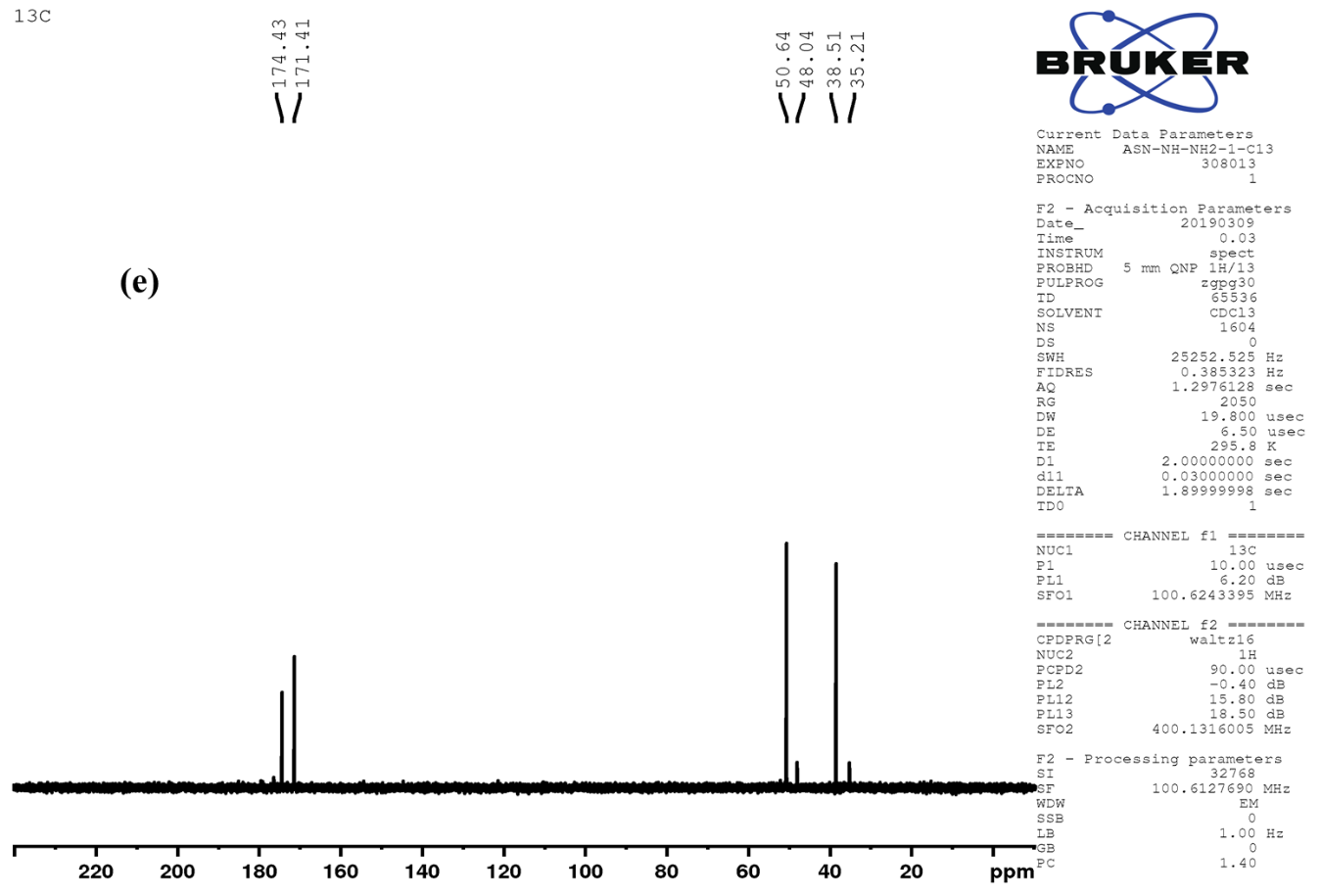


**Figure S2e.** The ^13^C-NMR spectrum of Asn-NHNH_2_.

All the signals in the ^1^H-NMR and ^13^C-NMR spectrum were successfully assigned in figure S2d. and S2e. ^1^H-NMR: (400 MHz, D_2_O-d_6_) δ (ppm): 3.6 (s, 1H), 2.5 (d, 2H). ^13^C-NMR: (400 MHz, D_2_O-d_6_) δ (ppm): 174.4, 171.41, 50.6, and 38.5.

**Complete compound 4 characterization data**


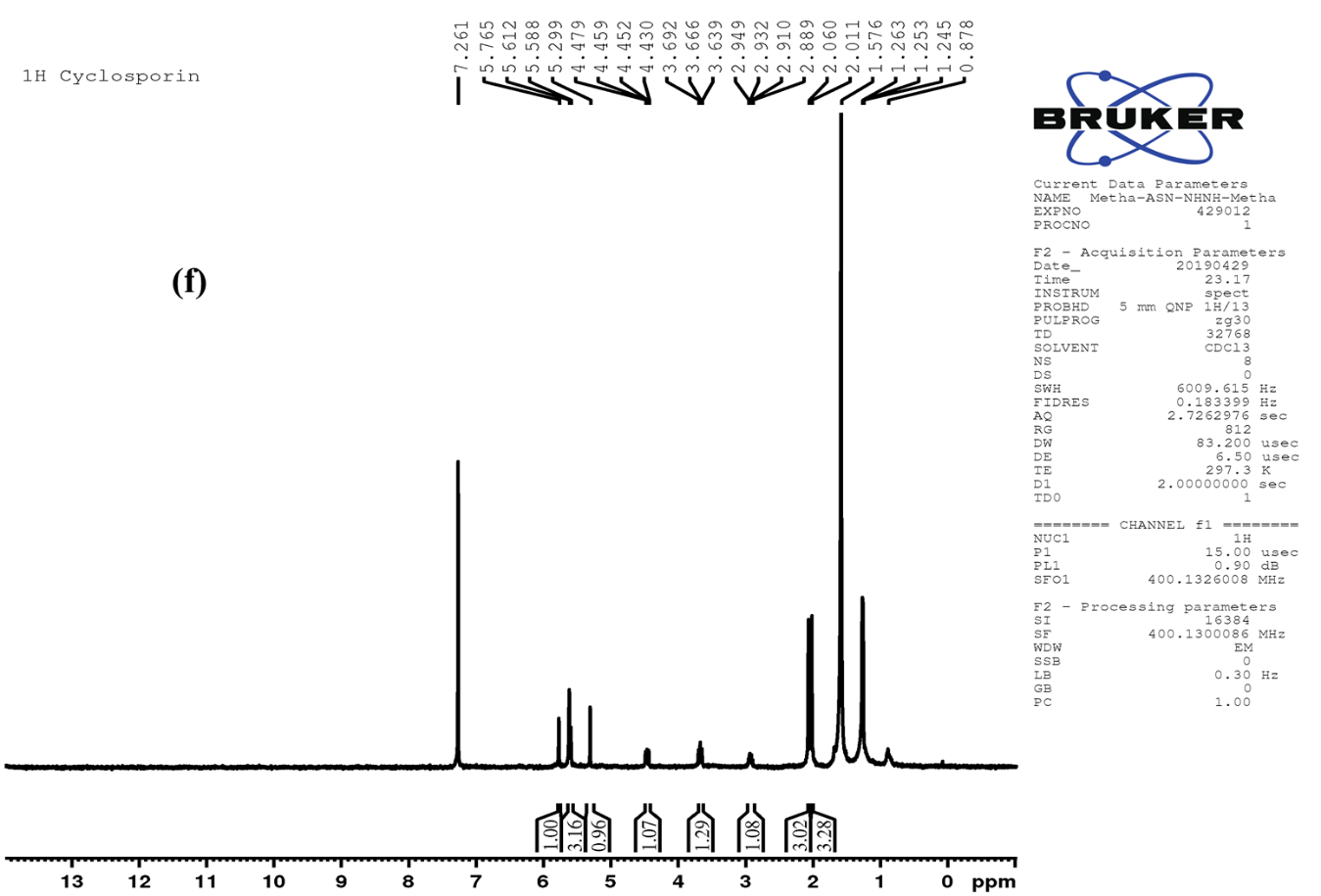


**Figure S2f.** The ^1^H-NMR spectrum of Metha-Asn-NHNH-Metha.

All the signals in the ^1^H-NMR were successfully assigned in figure S2f. ^1^H-NMR (400 MHz, CDCl_3_-d_6_) δ (ppm): 5.7 (s, 1H), 5.58-5.61 (d, 3H), 5.2 (s, 1H), 4.43-4.47 (m, 1H), 3.63-3.69 (m, 1H), 2.88-2.94 (m, 1H), 2.05-2.01 (d, 6H).

1. **Composition of the polymer synthesized.**

**Table S1.** Composition of the monomer and cross-linker used for PCIMPs synthesis

**Notr:** 100 mg of Fe3O4@APTMS-GA-Acrylate used in the preparation of PCIMPs. All polymerization solutions are 12 mL (TFE: H_2_O = 7: 3). AA=acrylamide. BAA= *N*-benzylacrylamide, ^a^EBAA= (*N,N*’-Ethylene bisacrylamide), and ^b^Metha-Asn-NHNH-Metha

1. **^1^H-NMR spectrum of reaction mixture formed by PAP/PCIMPs**

**Figure S3. ^1^H-NMR spectrum of the reaction mixture of starting material and ester formed by PAP/PCIMPs^66-78^**

1. **Affinity of PCIMPs toward papain solution was measured.**

Polymerization of acrylamide 8.4 mg ( 120 μmole ), *N*-benzylacrylamide 37.6 mg ( 240 μmole ), and *N,N*’-ethylenebisacrylamide 70.4 mg ( 420 μmole ) in 6 mL (H_2_O :TFE = 7 : 3 ) was performed to obtain PCIMPs with different amount of template. The Scatchard plots of these PCIMPs were used to obtain K_d_ value toward papain solution.

1. Template: PAP^66-78^, 5 mg, K_d_= 1330 nM

1. Template: PAP^66-78^, 2 fold amount of template, K_d_= 420 nM

(c) Template: PAP^66-78^, 3 fold amount of template, K_d_= 120 nM

1. Template: PAP^66-78^, 4 fold amount of template, K_d_= 102 nM
